# WNT7A Overexpression Inhibits Growth and Migration of Hepatocellular Carcinoma via the *β*-Catenin Independent Pathway

**DOI:** 10.1155/2019/3605950

**Published:** 2019-11-18

**Authors:** Lihui Lan, Wei Wang, Yue Huang, Chenghai Zhao, Xianmin Bu

**Affiliations:** ^1^Department of Hepatobiliary and Spleen Surgery, Shengjing Hospital, China Medical University, Shenyang, China; ^2^Department of Pathophysiology, College of Basic Medical Science, China Medical University, Shenyang, China; ^3^Department of Clinical Laboratory, Shanghai Pudong Hospital, Fudan University, Shanghai, China

## Abstract

*Background/Aims.* Hepatocellular carcinoma (HCC) is the lethal digestive cancer and the second leading cause of cancer death in men worldwide. Wnt7a, a 39Kd secreted glycoprotein composed of 349 amino acids, was reported to be related to various diseases. However, its role in HCC has not been studied yet. In this study, using gene expression data and clinical information obtained from the Oncomine and KMplot database, we acknowledged that WNT7A was underexpressed in HCC cancer tissue compared with normal tissue, and WNT7A underexpression was correlated with the decreased survival rate of HCC patients. The function of Wnt7a in cell viability, apoptosis, and migration was evaluated by biological behavior assay and molecular analysis. The findings revealed that WNT7A overexpression significantly restrained cell viability and migration while enhancing apoptosis. In addition, WNT7A overexpression promoted cell apoptosis by strengthening Caspase-3 activity and inhibited migration by downregulating EMT transcriptional factor Snail. Furthermore, the expression level of SKP2 was significantly downregulating in the WNT7A overexpression group. In conclusion, this study illustrated that overexpression of WNT7A inhibited cell viability and migration, which was likely attributed to the regulation of SKP2/P21.

## 1. Introduction

 Hepatocellular carcinoma (HCC) is the second leading cause of cancer death in men worldwide [1]. More than 0.7 million deaths from liver cancer occur per year, mostly because of the lack of early diagnosis and treatment. Despite the improvements of liver cancer treatment such as surgical resection, chemotherapy, and radiotherapy, the 5-year survival rate is dismal (below 20%) [2]. Over 80% of patients were diagnosed at a late stage when the tumor has grown and spread; thus, the local treatment was noneffective [3]. Researches on the molecular mechanisms of hepatocellular carcinoma led clinicians and investigators to concentrate on targeted therapy. To date, only two targeted therapies, sorafenib (an antiangiogenic agent and MAP kinase inhibitor) and regorafenib (a multikinase inhibitor), could increase overall survival [4, 5].

Wnt signaling pathway is an indispensable component of physiologic process referred to embryonic development and tissue homeostasis [6, 7]. It mainly exerts effects by initiating at least three types of Wnt signaling pathways: the canonical pathway (also named *β*-catenin dependent pathway), the calcium-dependent pathway (Wnt/Ca^2+^), and the planar cell polarity pathway (Wnt/PCP) [8]. Dysregulation of those pathways has been considered involved in the early stage of hepatocarcinogenesis [9].

Wnt7a, a member of Wnt protein family (one of 19 different Wnt proteins), is a 39Kd secreted glycoprotein composed of 349 amino acids. In humans, the encoding gene is located on chromosome 3P25.1 consisting of 4 exons and 3 introns (PMID: 31271226). Previous studies have demonstrated that the aberrant expression of Wnt7a exists in a variety of cancers. In ovarian carcinoma and endometrial carcinoma, the presence of Wnt7a led to the poor prognosis [10]. On the other hand, the restoration of Wnt7a could inhibit the proliferation in lung cancer and cervical cancer [11]. But, to our knowledge, no research on the correlation between Wnt7a activity and hepatocellular carcinoma is reported now.

In this study, we first evaluated the relationship between Wnt7a mRNA expression levels and overall survival of patients and worked out the Wnt7a expression profile changes in HCC tissues and tumor-derived cell lines. The roles of Wnt7a in growth, apoptosis, and migration were determined. Then, we tried to illustrate how Wnt7a exerted its influence on hepatocellular carcinoma. It appeared that low expression of Wnt7a was a key step during liver carcinogenesis. Those findings could be beneficial to the diagnosis or prognosis observation of liver cancer.

## 2. Materials and Methods

### 2.1. Chemicals and Reagents

The recombinant human Wnt7a was purchased from R&D System. WNT7A overexpression plasmids were from GeneChem. All antibodies were purchased from Abcam.

### 2.2. Cell Line and Culture

The human hepatocellular carcinoma cell lines HepG2, SMMC-7721, Hep3B, and Huh-7 were purchased from the Institute of Cell Bank (Shanghai, China). Hep3B and the remaining cell lines were cultured in Minimum Eagle Medium (MEM) and Dulbecco's Modified Eagle Medium (DMEM) containing 10% fatal bovine serum at 37°C with 5% CO_2_, respectively. Hep3B cells were serum starved for 2 h and then stimulated with 100 ng/ml of recombinant Wnt7a.

### 2.3. HCC Samples

Paraffin-embedded specimens that had been surgically removed from 33 patients with HCC, from January 1, 2013, to January 1, 2014, were collected. All samples were from patients diagnosed with hepatocellular carcinoma in this study, and both tumor tissues and adjacent nontumor tissues were included.

### 2.4. Immunohistochemistry

The 4 *μ*m sections were dehydrated using graded ethanol, inactivated for endogenous peroxidase with 3% hydrogen peroxide in PBS for 15 minutes at RT, and blocked with 1% bovine serum albumin in PBS for 30 minutes. Subsequently, the sections were incubated with monoclonal anti-Wnt7a antibody diluted 1 : 200 in PBS at 4°C overnight in a humidified chamber and then stained with goat anti-rabbit secondary antibody for 2 h at RT. Sections were then treated with 3,3′-diaminobenzidine (DAB) for approximately 10 minutes until color developed. Stained sections were observed by a light microscope. Fluorescent quantitation was performed using IPP 6.0 and the average integral optical density (IOD) (IOD/area) was used to represent expression level.

### 2.5. Western Blot Analysis

The proteins were extracted using radioimmunoprecipitation assay (RIPA) buffer on ice for 40 minutes. After centrifugation, the supernatant was harvested. The protein concentration was tested by BCA protein assay. Then, 25 *μ*g of total proteins was separated using 10% SDS-PAGE gels. The proteins were then transferred onto polyvinylidene fluoride (PVDF) membranes at 50 V for 1.5 h at 4°C. After protein transfer, the membranes were blocked with 5% BSA in TBST for 2 h and incubated overnight with primary antibodies at 4°C. After being incubated with the HRP-conjugated specific secondary antibody for 2 h at room temperature, the blot was revealed with enhanced chemiluminescence western blotting detection reagent. *β*-Actin was used as the loading control for normalization.

### 2.6. Apoptosis Detection

To assess apoptosis, Annexin V/PI Apoptosis Detection Kit was used. Hep3B cells were digested and harvested and, then, spun for 5 minutes at the speed of 1200 rpm. Isolated single cells were washed twice with PBS. Finally, cells were stained with Annexin V and propidium iodide as indicated by protocol. Flow cytometry was performed using BD Accuri C6 Plus.

### 2.7. MTT Assay

The Hep3B cells transfected with WNT7A overexpression plasmids for 24 h were seeded into 96-well plates in triplicate at 3 × 103 cells. The Hep3B cells were subjected to recombinant human Wnt7a (rWnt7a) treatment (100 ng/ml) for the indicated time. At the end of experiments, 20 *μ*l MTT (5 mg/ml) was added to each well and the cells were incubated at 37°C for an extra 4 h. The supernatant was then discarded, 150 *μ*l DMSO was added to each well to dissolve formazan crystals, and cells were incubated for 10 minutes. The optical density was measured at 490 nm.

### 2.8. Transwell Migration Assay

The Hep3B cells were transferred into the upper chamber (1 × 10^5^/chamber) of transwell (PC membrane with 8.0 *μ*m pore size) in MEM, and the lower chambers were supplemented with MEM containing 20% FBS as chemoattractant following treatment with rWnt7a (100 ng/ml). After culture for 36 h, the upper chambers were fixed with paraformaldehyde and stained with 0.1% crystal violet. Five fields of view were randomly selected to count cells using light microscopy (200×).

### 2.9. Immunofluorescence

Briefly, Hep3B cells were fixed with 4% paraformaldehyde for 10 minutes and permeabilized using 0.5% Triton X-100 for 15 minutes. Then, the cells were blocked with 1% bovine serum albumin for 30 minutes at 37°C, and they were incubated with mouse monoclonal *β*-catenin antibodies at 4°C overnight. The slides were washed 3 times with PBS and incubated with goat anti-mouse secondary antibodies at RT for 1 h. Finally, the slides were stained with DAPI for 10 minutes and then washed with PBS. Fluorescence images were captured by fluorescence microscope.

### 2.10. Survival Analysis

Survival analysis for hepatocellular carcinoma patients with different Wnt7a mRNA expression levels was performed using the KM plot database (http://www.kmplot.com).

### 2.11. Statistical Analysis

Statistical analysis was performed to evaluate the statistical significance of the quantitative data. The difference between two groups was analysed by Student's *t*-test. *p* value less than 0.05 was considered as statistically significant.

## 3. Results

### 3.1. Wnt7a Underexpression Is Correlated with the Decreased Patient Survival

**Figure 1:**
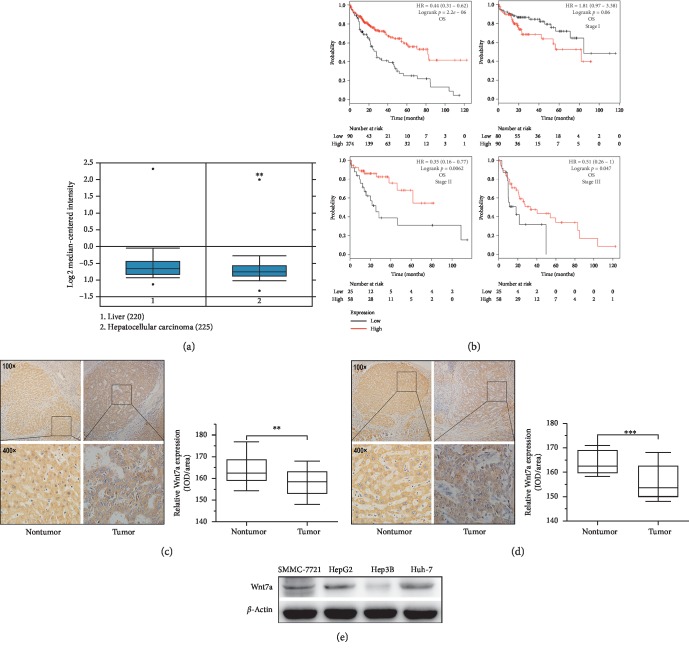
Decreased mRNA expression of WNT7A in hepatocellular carcinoma and association with poor patient survival. (a) Oncomine database analysis with the mRNA expression level of WNT7A in normal and tumor tissues. (b) Kaplan–Meier plots of OS and WNT7A expression on different stages using the KM plot database (http://www.kmplot.com). (c) Representative images from IHC analysis of Wnt7a expression in clinical Stage II HCC tissues and paracarcinoma tissues. (d) Representative images from IHC analysis of Wnt7a expression in clinical Stage III HCC tissues and paracarcinoma tissues. (e) Wnt7a expression in different HCC cell lines measured by western blot analysis. ^*∗*^*p* < 0.05; ^*∗∗*^*p* < 0.01; ^*∗∗∗*^*p* < 0.001.

In order to understand the potential role of Wnt7a in hepatocellular carcinoma, we analysed the correlation between WNT7A RNA expression levels and overall survival in HCC. WNT7A was found to be underexpressed in HCC (*p*=0.003) (Figure 1(a)) using Oncomine gene expression database (http://www.oncomine.com) [12]. The survival analysis indicated that WNT7A underexpression was associated with worse overall survival (*p*=2.2*E* − 06) (Figure 1(b)). All the overall survival analysis including stage I, stage II, and stage III was based on the 364 hepatocellular carcinoma patients from the KM plot database. The *p* value in stage I was not practically significant (*p* > 0.05) but significant in stage II (*p*=0.0062) and stage III (*p*=0.047). That meant Wnt7a might be a prognostic tool for hepatocellular carcinoma and more possibly affected the late stage. These data suggest that Wnt7a plays a protective role in the development of hepatocellular carcinoma.

### 3.2. Wnt7a Expression Is Decreased in Liver Cancer Specimens but Different in HCC Cell Lines

Next, our team quantified Wnt7a protein levels and subcellular localization to supplement the RNA-based WNT7A expression level. We first localized the Wnt7a levels using immunohistochemistry across 33 patient-derived liver cancer tissues. And we found Wnt7a protein permeated at cytoplasm with a decreased signal in cancer tissues compared to paracarcinoma tissues in 21 specimens (Figures 1(c) and 1(d)). Finally, we examined the Wnt7a protein level on the tumor-derived cell lines (SMMC-7721, HepG2, Hep3B, and Huh-7) by Western blot analysis. Wnt7a was not detected in tumorigenic cell line Hep3B but moderately expressed in SMMC-7721, HepG2, and Huh-7 (Figure 1(e)). Therefore, we chose to focus on Hep3B cells for further experiments.

### 3.3. Wnt7a Inhibits Cell Viability but Promotes Apoptosis of HCC Cells

**Figure 2:**
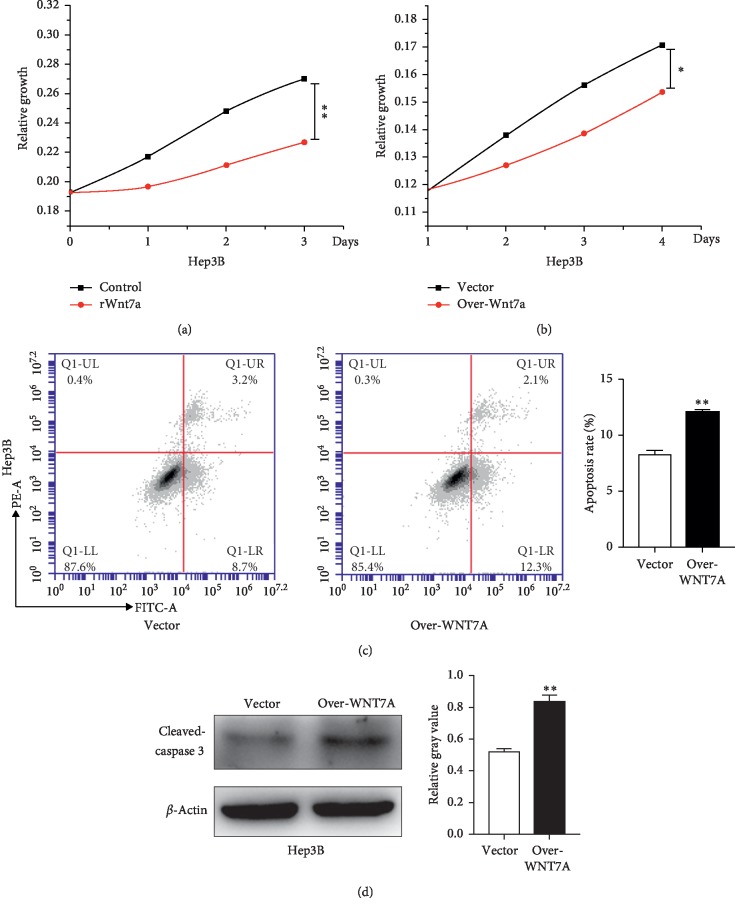
Wnt7a represses the viability and promotes apoptosis of HCC cells. (a) MTT assay was used to determine the growth characteristics of Hep3B cells cultured in media containing rWnt7a. (b) MTT assay was used to determine the growth characteristics of Hep3B cells with WNT7A overexpression. (c) Apoptosis was detected using FCM. The numbers showed the percentages of each fraction. (d) Western blot analysis images of cleaved-caspase-3 expression in Hep3B cells with WNT7A overexpression. ^*∗*^*p* < 0.05; ^*∗∗*^*p* < 0.01.

Because Wnt7a underexpression was found in HCC specimens and correlated with worse overall survival, we assumed that downregulation of Wnt7a was a component factor during carcinogenesis. For the sake of further investigating the roles of Wnt7a in this process, we designed several experiments to ascertain the effects that Wnt7a might have on growth of HCC cells. For this purpose, Hep3B cells with Wnt7a low expression were stimulated with recombinant human Wnt7a protein (rWnt7a) in an exogenous manner with 100 ng/ml for 72 h, and subjected to MTT assay, simultaneously. As shown in Figure 2(a), the addition of rWnt7a into Hep3B cells remarkably reduces the cell viability compared with untreated cells. On the other hand, Hep3B cells were transiently transfected with WNT7A overexpression plasmids against control and then also subjected to MTT assay for the indicated periods of time. It showed similar results as presented in Figure 2(b). These results suggest that Wnt7a represses growth of liver cancer Hep3B cells.

Finally, Annexin V-FITC/PI flow cytometry was used to detect apoptosis on Hep3B cells; as presented in Figure 2(c), the apoptosis rate was increased after overexpression of WNT7A. We further detected caspase-3 activity using anti-cleaved-caspase-3 antibody. Consistent with Annexin V/propidium iodide staining, the caspase-3 activity was increased in Hep3B (Figure 2(d)). Taken together, all these observations show that Wnt7a inhibits growth and promotes apoptosis in liver cancer cells.

### 3.4. Wnt7a Protein Inhibits Migration of HCC Cells by Downregulating EMT

**Figure 3:**
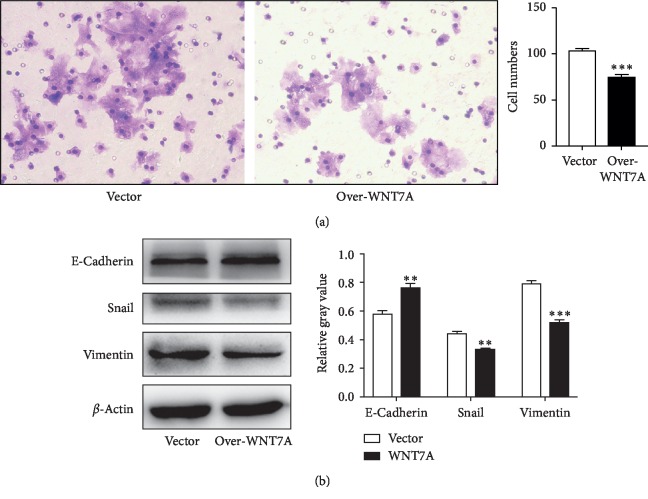
Wnt7a inhibits migration of Hep3B cells by regulating EMT. (a) Transwell migration assay was performed on Hep3B cells with WNT7A overexpression. (b) Western blot analysis for EMT protein levels in Hep3B with WNT7A overexpression for 48 h. ^*∗*^*p* < 0.05; ^*∗∗*^*p* < 0.01; ^*∗∗∗*^*p* < 0.001.

To understand other functional significance of Wnt7a in HCC cell lines, we wondered whether Wnt7a exerted influence on migration. As mentioned above that Wnt7a might have more effect on the late stage than the early stage, we postulated that migration would also be suppressed by the presence of Wnt7a. To verify this, transwell assay on Hep3B cells with WNT7A overexpression was conducted and estimated after 48 hours. Consistent with our speculation, the results manifest that migration ability of cells is reduced compared with control cells (Figure 3(a)).

Next, epithelial-mesenchymal transition (EMT) related genes including E-cadherin, vimentin, and Snail were detected by immunoblotting. Hep3B overexpressed with WNT7A did show an increased expression of epithelial cell marker (E-cadherin) and decreased expression of mesenchymal cell marker (Vimentin) and EMT transcriptional factor (Snail) (Figure 3(b)). These data suggested that loss of Wnt7a might be a risk factor for the development of hepatocellular carcinoma.

### 3.5. Wnt7a Represses Hepatocellular Carcinoma via the *β*-Catenin Independent Pathway

**Figure 4:**
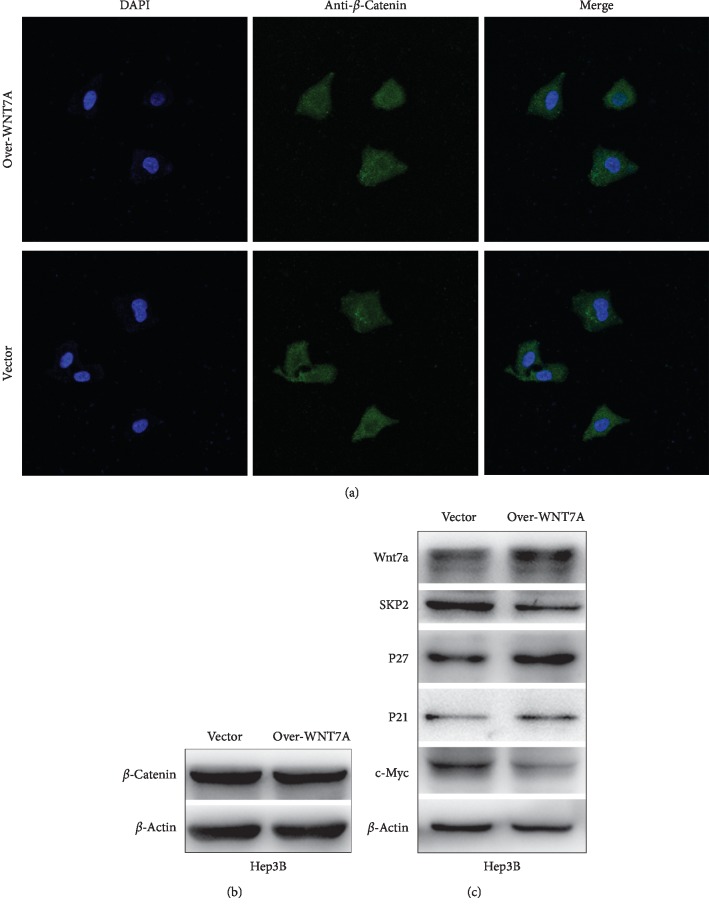
SKP2/P21 is involved in the suppressive effect of Wnt7a on growth and migration of HCC. (a) Expression of *β*-catenin was detected by immunofluorescence staining after transfection. Merged pictures were overlays of both *β*-catenin green signals and nuclear staining by DAPI (blue) (magnification: ×400). (b, c) Representative western blot analysis images for the indicated proteins in Hep3B cells with WNT7A overexpression.

*β*-Catenin is a protooncogene, and mutation of this gene is universal in hepatocellular carcinoma [13, 14]. To confirm whether *β*-catenin also participated in Wnt7a-mediated hepatocarcinogenesis, we detected *β*-catenin levels by immunoblotting and immunofluorescence staining in Hep3B cells (Figure 4). The expression level of *β*-catenin is similar in both control and WNT7A overexpression groups (Figure 4(b)). Immunofluorescence staining showed that WNT7A overexpression does not significantly increase the accumulation and nuclear translocation of *β*-catenin (Figure 4(a)). These data suggest that tumor-suppressive effects of Wnt7a are not mediated by *β*-catenin pathway.

To further investigate the molecular mechanism by which Wnt7a plays its tumor-suppressive role, we detected the expression level of antioncogene P21. Western blotting showed that P21 expression was increased in WNT7A overexpression group. S-phase kinase-associated protein 2 (SKP2), a substrate recognition component of ligase complex also considered as a oncogene during tumorigenesis [15], mediates the ubiquitination and degradation of CKIs. Previous research indicated that Wnt7a could inhibit tumor by regulating SKP2/P21 signaling. To verify whether Wnt7a upregulates P21 through SKP2. SKP2 expression level was measured by Western blot in Hep3B cells overexpressed with WNT7A. Findings showed that overexpression of WNT7A significantly downregulated the SKP2 expression level (Figure 4(c)). To testify whether Wnt7a really downregulated SKP2, another target of SKP2, P27, was also detected by Western blotting. Within our expectation, P27 was upregulated as well. On the contrary, the oncogene c-Myc was downregulated in WNT7A overexpression group. These findings suggested that Wnt7a upregulated P21 and P27 by inactivating SKP2.

## 4. Discussion

According to the multifactor model of hepatocellular carcinoma, there are various confirmed evidences about molecular pathways that participate in liver cancer transformation [16, 17]. Classically, the Wnt signaling cascade was regarded as a developmental pathway, but its dysregulation was commonly associated with many diseases, especially cancer [18]. The fact that *β*-catenin, a critical molecular of Wnt-canonical pathway, accumulated in cytoplasm was one of evidences that Wnts activated the Wnt signaling. In fact, activated *β*-catenin translocated into nuclear would increase the expression of target genes and promote progression in HCC cells [19].

In our study, we found that Wnt7a was underexpressed in 21 liver cancer tissues, out of 33 cases in total. This expression level was highly consistent with mRNA expression levels from cBioPortal for Cancer Genomics. According to the data of this database, the mRNA upregulation of Wnt7a only accounted for 0.3% in all 366 HCC cases. As far as we acknowledged, there was no previous study regarding biological activity of Wnt7a in the hepatocarcinogenesis. Nevertheless, other cancer models already provided multiple valuable evidences for us. For instance, it has been demonstrated that Wnt7a expression was reduced owning to the methylation in clear cell renal cell carcinoma [20]. Furthermore, it was determined that Wnt7a was underexpressed in the cervical cancer cells and inhibited proliferation [9]. Wnt7a was also inactivated in lung cancer, and restoration of Wnt7a could accelerate cell senescence [21].

On the other hand, Wnt7a was found highly expressed in the bladder cancer and pancreatic ductal adenocarcinoma [22, 23], and Wnt7a overexpression in these two cancers not only promoted metastasis but also predicted poor prognosis. Wnt7a overexpression was also found in ovary cancer [24] and promoted tumor by inducing activation of CAFs [25]. In addition to extremely different expression levels in multitype tumors, Wnt7a ligand could trigger both canonical and noncanonical pathways relying on the receptors it interacted with. As we previously reviewed, binding to FZD7, FZD9, and FZD10, Wnt7a could initiate noncanonical pathway [26]. It could also activate canonical pathway by interacting with FZD5 [24].

Abnormal cell growth is one of the markers in tumor development. We found that restoration of Wnt7a expression could inhibit cell growth in Hep3B. At this point, a previous study also revealed that Wnt7a obviously reduced the cell viability of cervical cancer [9], which shows no difference from our results. The migration ability of liver cancer cells is the primary factor that results in decreased survival rate of HCC patients [27]. Using transwell assay, we revealed that WNT7A overexpression inhibited tumor cell migration. EMT is associated with tumor migration, invasion, and metastasis [28]. The results showed that WNT7A overexpression could inhibit EMT by downregulating EMT transcriptional factor Snail. A new finding in this study is that WNT7A overexpression can promote tumor cell apoptosis. At the molecular level, the Caspase-3 activity remarkably increased in Hep3B with WNT7A overexpression.

SKP2 plays a carcinogenic role by the ubiquitylation and degradation of P21, P27, P57, and E-Cadherin. SKP2 overexpression has been seen in multiple human cancers such as prostate cancer [29], pancreatic cancer [30], breast cancer [31], and melanoma [32]. When cells stimulated with extracellular oncogenic signals, Wnt7a was secreted. The secreted Wnt7a inactivated SKP2 leading to upregulation of P21, which inhibits the growth of hepatocellular carcinoma. Previous studies have revealed that upregulation of SKP2 could promote the migration and invasion of HCC cells by the degradation of P21 and P27 [33]. Our experiment results shared the similar view on this point. Therefore, Wnt7a can affect cell growth and migration by regulating SKP2/P21.

In conclusion, Wnt7a, as an antioncogene in hepatocellular carcinoma, effectively regulated Wnt target genes that are important for HCC growth and progression. The correlation between low Wnt7a expression level and reduced overall survival of HCC patients indicates that Wnt7a may be a valuable prognostic marker. However, the specific mechanisms of Wnt7a regulation of tumor-suppressive signaling in HCC required further investigation.

## Data Availability

The data used to support the findings of this study are available from the corresponding author upon request.
